# Study on the Roasting Process of Guisha Limonite Pellets

**DOI:** 10.3390/ma15248845

**Published:** 2022-12-11

**Authors:** Chuang Zhang, Xiaolei Zhou, Lei Gao, Haoyu Fang

**Affiliations:** Faculty of Metallurgical and Energy Engineering, Kunming University of Science and Technology, Kunming 650093, China

**Keywords:** bentonite, pellet ore, pure limonite, response surface, DOE

## Abstract

In this paper, a pelletizing method has been researched to enhance the subsequent iron-making process applying Guisha limonite, with advantages including large reserves and low price. The purpose is to provide an alternative for the sinter, thus reducing the greenhouse gas emission during the iron-making process. The response surface method is used to optimize the experimental design of the pelleting process. A multivariate regression model for estimating the compressive strength of pellets was developed using Box–Behnken experimental methodology, where the relevant factors were the roasting temperature, pellet diameter, and bentonite content. The maximum influencing factors of each experimental design response are determined using analysis of variance (ANOVA). Under optimum conditions, the compressive strength of pure limonite pellets is 2705 N, similar to the response goal value of 2570.3 N, with a relative error of 5.20%. Since the high-grade iron ore resources are depleted, the comprehensive utilization of ore resources is becoming increasingly important. The aim of this paper was to provide a valuable technical foundation for lignite pellet-roasting processes in the iron and steel industries, since steel companies is increasing its imports of Guisha limonite.

## 1. Introduction

The iron and steel industry is one of the significant contributors to CO_2_ emissions and faces the challenge of a deficiency in high-grade ores. Sintering is an important preprocess before iron making in the blast furnace. However, the sintering process has a long flow, with enormous greenhouse gas emissions and high energy consumption [1,2,3]. Pellets can improve the breathability in the blast furnace, which increases the reduction process’s efficiency and reduces greenhouse gas emissions. Additionally, the application of pellets can increase the tolerance of low-grade ores and thus is noticed as a cost-saving technique compared to the application of sinter [4,5,6].

Guisha limonite is mainly produced in Laojie Province as a typical lean ore, with abundant reserves and affordable pricing. Guisha limonite (2Fe_2_O_3_·H_2_O) is a kind of hydrous iron oxide ore after weathering and has low iron content. Although significant progress was made in applying Guisha limonite in sinter production, research on the production of Guisha limonite pellets is still quite limited [7]. Because of the low sintering strength and high reduction pulverization rate of Guisha limonite, it is not easy to further improve the sintering process with Guisha limonite as raw material. To solve this problem, industries in various countries have increased the development and utilization of limonite pellets. With increased Guisha limonite imports in iron and steel companies, research on Guisha limonite pellets has become a hot topic recently. The consolidation mechanism of Guisha limonite can be generally divided into three steps: (1) Fe_2_O_3_ grain development, (2) expansion of the grain, and (3) interconnection of the grains into a complete consolidation, which is commonly referred to as solid-phase consolidation. Many experiments have revealed that Fe_2_O_3_ crystallization is a process that progresses from primary crystals to developed crystals and finally to interconnected crystals, with primary crystals typically forming at about 1150–1200 °C. At 1220–1250 °C, developed crystals form; above 1280 °C, interconnected crystals form [8,9,10]. The crystallization process is critical to the compressive strength of pellets [11]. As limonite is a highly crystalline water ore, the desorption of crystalline water occurs at 200–500 °C, which increases the internal stress of the pellets and causes pellet blast [12,13]. At the same time, during the hardening process of the pellets, the crystal water converts into steam under high temperature, escaping from the pellets, resulting in the generation of cracks and pores. As a result, the process reduces the compressive strength of the pellets. The motivation behind this study was to optimize the roasting conditions of low-grade limonite to produce pellets with high-quality characteristics. The results may provide suggestions for the utilization of limonite.

In the past, research on the compressive strength of pellets mostly used single-factor analysis or orthogonal testing to qualitatively describe the impact factor of each parameter. However, the influences of the interaction of various aspects have not been considered [14]. The predictability is poor because the number of trials is small [15,16]. The response surface methodology is utilized in this paper to optimize the experimental design of the preparation of limonite pellets [17,18,19]. The experimental factors affecting the strength of limonite pellets were studied systematically. The optimal roasting process was obtained, including the bentonite content, roasting temperature, and pellet diameter, which improved pellet thermal burst temperature, compressive strength, and so on [20,21]. The attainment of complete resource utilization and pollutant emission reduction is projected to provide a valuable technical foundation for the roasting process of limonite pellets in the iron and steel industries.

## 2. Materials and Methods

**Figure 1:**
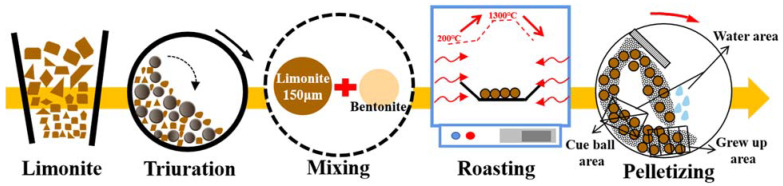
Limonite pellet-roasting process.

**Figure 2:**
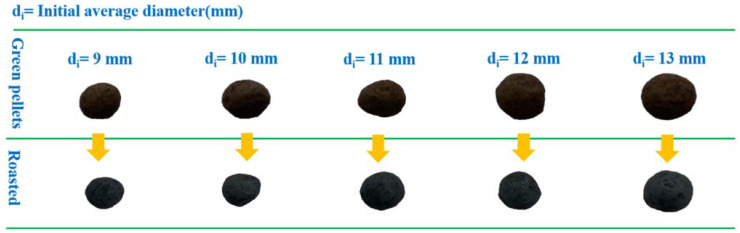
Different sizes of green pellets and roasted pellets.

A ball mill was used to grind Guisha limonite for 72 h prior to this experiment, and an automatic screening machine was used to complete the screening. We used 200-mesh Guisha limonite powder (200 g) and combined it with bentonite in a specific proportion (0.8wt%, 1.0wt%, 1.2wt%, 1.4wt%, 1.6wt% of the mineral powder’s mass). Distilled water (3.0wt%) was added equally to the mineral powder for wetting. Distilled water (7.0wt%) was sprayed to produce pellets with a diameter of 11–15 mm. The pellets were dried for 1 h in a drying oven at 110 °C to eliminate moisture and volatiles for subsequent use. Figure 1 depicts the experimental procedure, where the experiment was carried out in 75 groups. The details of the experimental design are shown in Table 1. During the experiments, 20 pellets from each group were placed in corundum crucibles and subsequently placed in a resistance furnace for calcination in an air atmosphere. After natural cooling of the samples in air, we tested the compressive strength of the pellets according to GB/T14201-93, namely “Determination of Compressive Strength of Iron Ore Pellets” [14]. The change in appearance of the pellet samples during the roasting process is shown in Figure 2.

## 3. Results and Discussion

### 3.1. Experimental Materials

**Figure 3:**
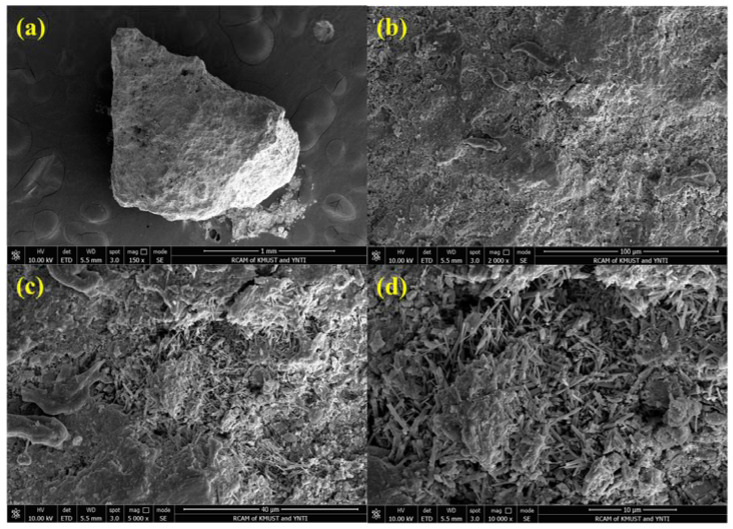
SEM of Guisha limonite: (**a**) 150×, (**b**) 2000×, (**c**) 5000×, (**d**)10,000×.

**Figure 4:**
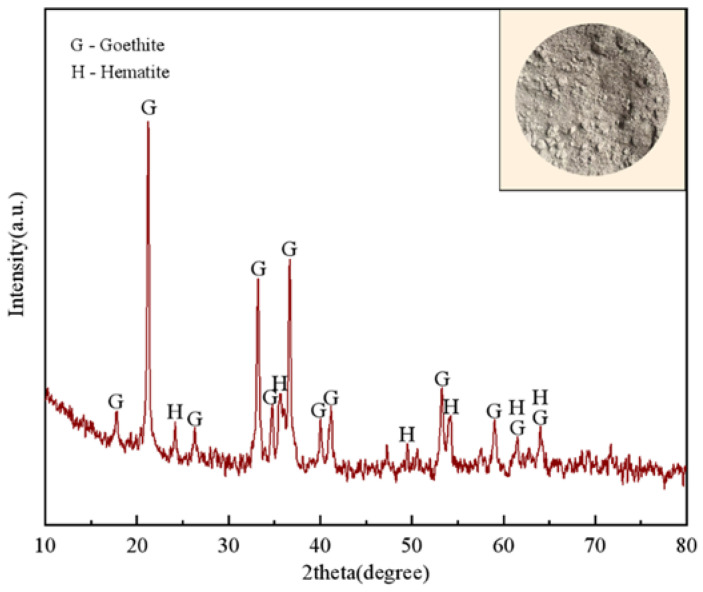
XRD pattern of Guisha limonite.

The Guisha limonite utilized in this experiment has a rough surface, a loose and porous structure, and small crystals. It is a typical highly crystalline ferrihydrite with a morphology resembling seafloor coral and a typical limonite crystalline condition. This largely determines that it has the characteristics of high porosity, strong water absorption, high wetting capacity, and easy melting. An SEM image of limonite is shown in Figure 3. Guisha limonite has 54.67% total iron concentration, making it a relatively low-grade mineral. The mass fraction of SiO_2_ sourced from gangue is 4.04%. It has a large variety of impurities, but the amount is minimal, and 14.82% of it is total moisture content. The different wettability of the raw materials mainly causes the difference in the growth of green pellets. When the raw material has good wettability, the water will form a film that adheres tightly to the pellet’s surface. After colliding with another pellet, a liquid bridge is formed, and the two pellets start to bond under the action of surface tension, which promotes growth. It can be found from the SEM image that the limonite particles of Guisha limonite sample are granular, with a rough surface and loose and porous structure. Guisha limonite has strong water absorption, and the wetting capacity of mineral powder is large. This means that limonite has strong surface hydrophilicity in the process of pelletizing, forming a more liquid film, which tends to bond more iron ore particles, which is beneficial to the growth of pellets. The Guisha limonite samples used in this study were from A certain Iron and Steel company in Yunnan Province, the chemical composition of which is shown in Table 2. X-ray diffraction was used to describe the material, and the limonite XRD pattern is displayed in Figure 4. The pattern revealed that FeOOH, which contains a significant amount of crystal water, is the primary mineral form of limonite. The bentonite selected in the experiment contains 85.97% montmorillonite, the montmorillonite content is high, and the physical and chemical properties of the bentonite are also good. The data are shown in Table 3.

**Figure 5:**
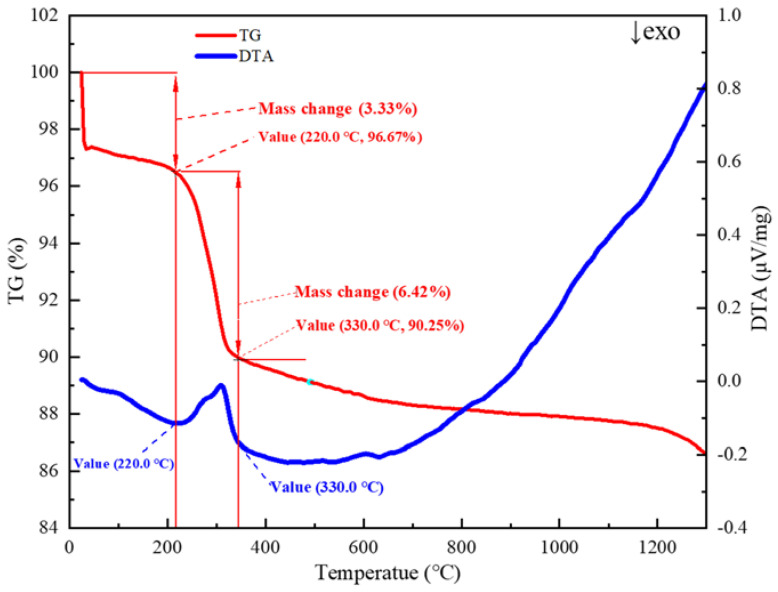
TG-DTA curve of limonite roasting process.

Crystal water desorption is a critical step in the limonite roasting process, primarily affected by oxidized pellets’ quality. The adsorbed water and crystal water of Guisha limonite will be lost during the roasting process as the material temperature rises. Figure 5 depicts the thermal–thermogravimetric (TG-DTA) curve of Guisha limonite samples, showing that the limonite has a noticeable weight loss before 220 °C and the initial weight loss is 3.33%. The weight loss of the sample is primarily caused by the loss of primarily surface moisture when the temperature is less than 220 °C, based on thermodynamics. When the temperature rises above 220 °C, the crystal water in the minerals begins to evaporate. When the temperature was raised to 220 °C, the limonite decomposed violently, resulting in an endothermic peak in the DTA curve, as shown in Equation (1).

(1) $${\mathrm{nFe}}_{2}O_{3}\cdot {\mathrm{mH}}_{2}O\to {n\gamma \text{-}\mathrm{Fe}}_{2}O_{3}+{\mathrm{mH}}_{2}O$$

At this stage, only the conversion of ${\mathrm{nFe}}_{2}O_{3}\cdot {\mathrm{mH}}_{2}O$ to ${\gamma \text{-}\mathrm{Fe}}_{2}O_{3}$ takes place; no lattice transformation occurs. However, ${\gamma \text{-}\mathrm{Fe}}_{2}O_{3}$ is not stable. When the temperature exceeds 330 °C, the breakdown of limonite ceases. Since the ${\gamma \text{-}\mathrm{Fe}}_{2}O_{3}$-phase is unstable, as shown in Equation (2), the crystals will rearrange at high temperatures [22].

(2) $${\gamma \text{-}\mathrm{Fe}}_{2}O_{3}\to {\alpha \text{-}\mathrm{Fe}}_{2}O_{3}$$

**Figure 6:**
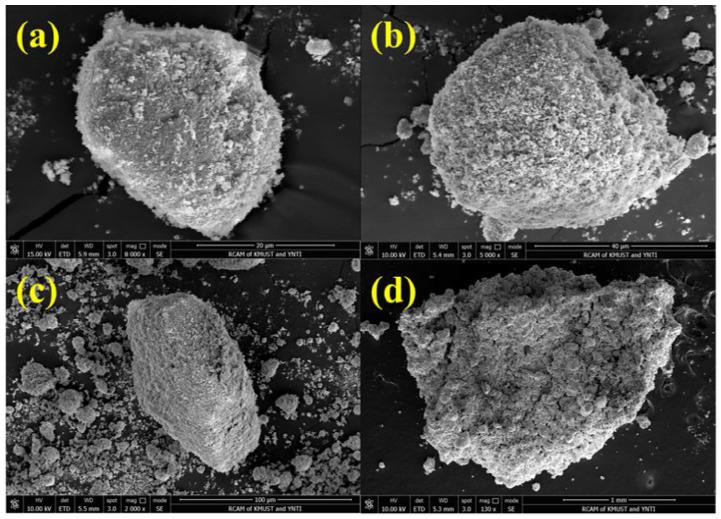
SEM of the evolution of limonite pellets at different temperatures: (**a**) 0 °C, (**b**) 200 °C, (**c**) 800 °C, (**d**) 1250 °C.

${\gamma \text{-}\mathrm{Fe}}_{2}O_{3}$ is converted into ${\alpha \text{-}\mathrm{Fe}}_{2}O_{3}$, and when the temperature is too high, part of the cations directly diffuse into the second stage, i.e., ${\mathrm{nFe}}_{2}O_{3}\cdot {\mathrm{mH}}_{2}O$ is transformed into ${\alpha \text{-}\mathrm{Fe}}_{2}O_{3}$.The sample’s quality gradually deteriorated as the temperature rose, and the crystal water left in the minerals was lost. When the temperature is elevated to 1300 °C, the sample’s weight loss can exceed 13%. Figure 6 depicts the microstructure evolution of limonite pellets at various temperatures. It is clear that as the temperature rises, the pores on the surface of the pellets gradually expand due to the removal of crystal water.

### 3.2. Single-Factor Test

#### 3.2.1. Influence of Roasting Temperature on Compressive Strength of Pellets

**Figure 7:**
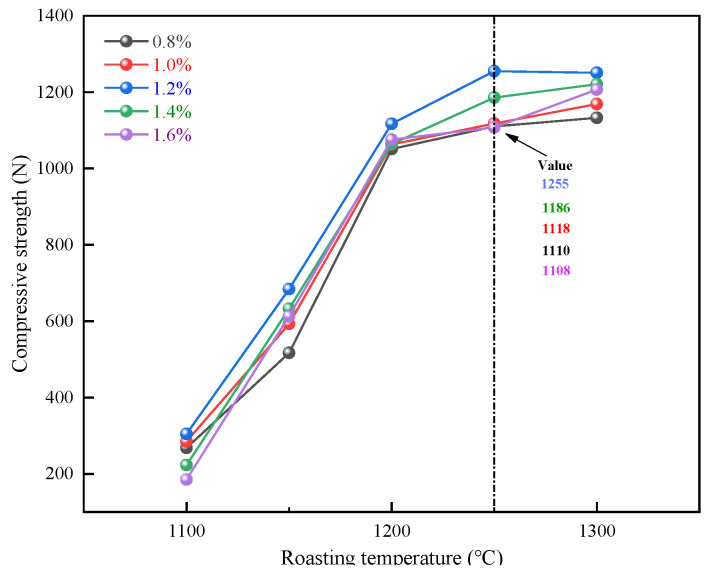
Effect of roasting temperature on the compressive strength of pellets.

**Figure 8:**
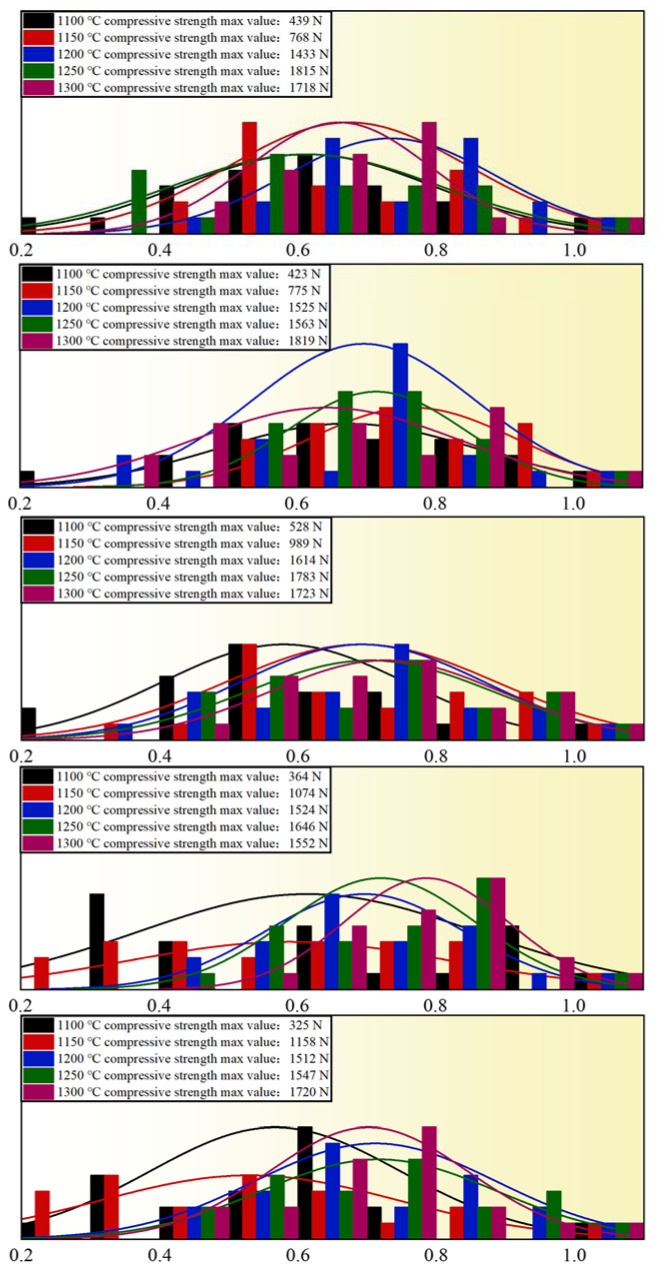
Normality test of pellet compressive strength under the influence of roasting temperature.

The diameter of pellets was controlled to be 9–13 ± 0.5mm, and the addition of bentonite was 0.8wt%, 1.0wt%, 1.2wt%, 1.4wt%, and 1.6wt%. The experiments were carried out in 25 groups to study the effect of roasting temperature on the compressive strength of pellets, with 20 pellets for each group. Figure 7 depicts the experimental outcomes. The normality test and linear analysis were carried out for 25 groups of experimental data. The analysis results are shown in Figure 8. The effect of roasting temperature on pellet compressive strength is substantial. When the temperature is between 1100 °C and 1250 °C, the compressive strength of pellets increases in a positive relationship with the roasting temperature, and the strength is virtually the same between 1250 °C and 1300 °C. As a result, the ideal roasting temperature for pure limonite pellets is around 1250 °C 1300 °C. This finding is because the strength of the pellets is mainly determined by the iron ore’s oxidative recrystallization process. The volume of the pellets will alter during the crystallization process. The higher the degree of crystallization, the faster the pellets expand as the temperature rises. It generates fissures in the pellets, diminishing their strength, and the effect on strength becomes more evident as the temperature rises.

#### 3.2.2. Influence of Roasting Temperature on Compressive Strength of Pellets

**Figure 9:**
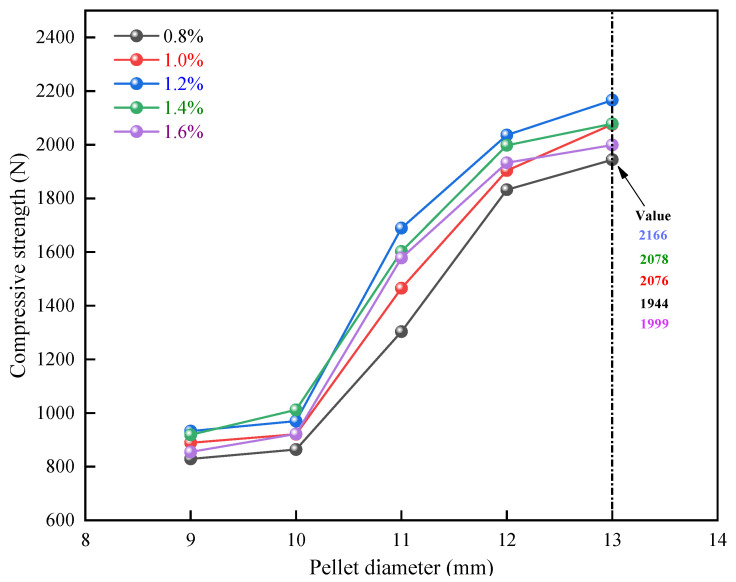
Effect of pellet diameter on the compressive strength of pellets.

**Figure 10:**
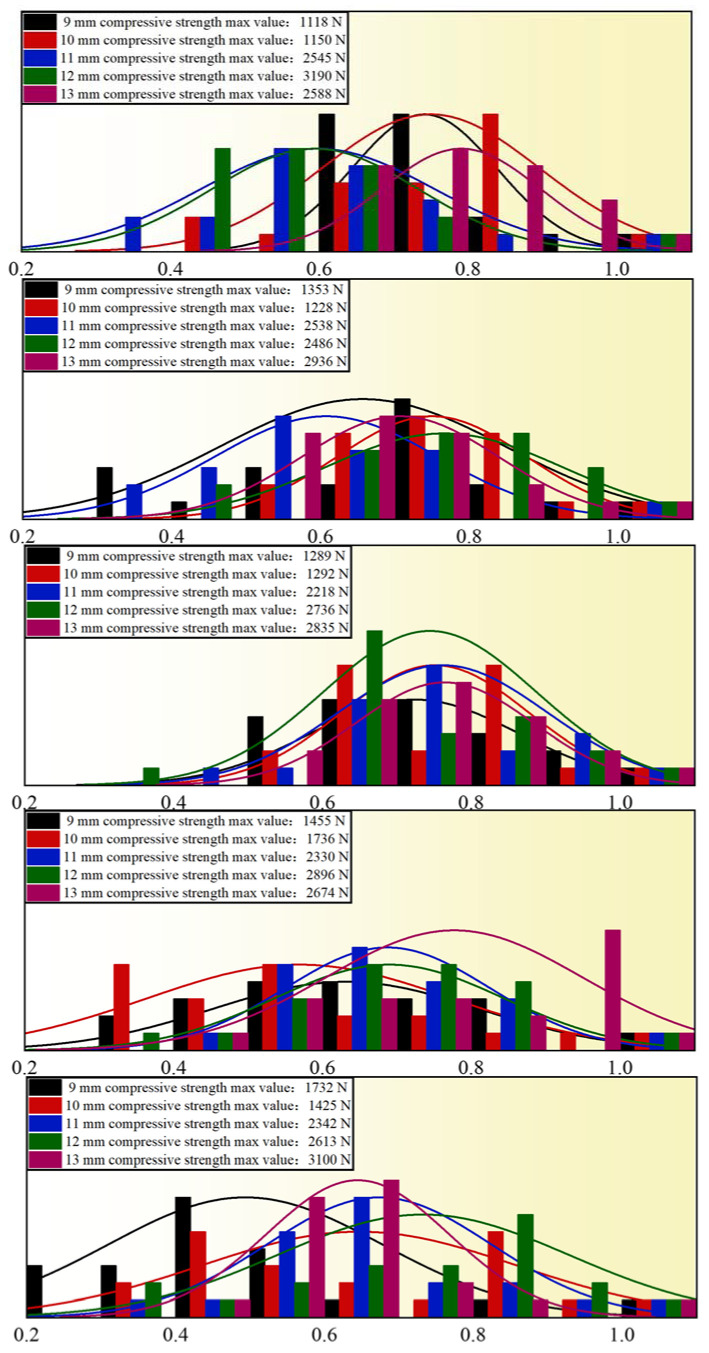
Normality test of pellet compressive strength under the influence of pellet diameter.

To investigate the influences of pellet diameter on compressive strength, the bentonite addition amount was regulated at 0.8wt%, 1.0wt%, 1.2wt%, 1.4wt%, and 1.6wt%, and the calcination temperature was 1250 °C. The experiments were carried out in 25 groups, with 20 pellets for each group. Pellets with diameters ranging from 9 mm to 13 mm were used for the compressive strength test. Figure 9 depicts the experimental findings. For 25 groups of experimental data, the normality test and linear analysis were performed. Figure 10 illustrates the analysis results. The compressive strength of the pellets does not vary much within a diameter range of 9–10 mm. It shows a growing tendency with the rise of the pellet diameter within the diameter of 10–13 mm, and the range exceeds 1000 N. This demonstrates that pellet diameter has a significant influence on pellet compressive strength. However, if the pellet diameter is too large, the unit heat consumption will be excessive, the oxidation will be insufficient, and the roasting and chilling time will be extended. As a result, the ideal pellet diameter is around 13 mm.

#### 3.2.3. Influence of Bentonite Addition on Compressive Strength of Pellets

**Figure 11:**
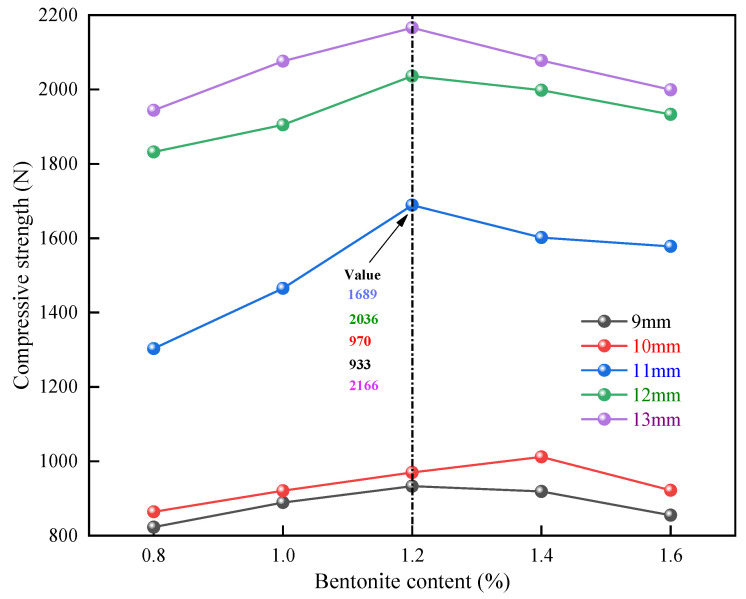
Effect of bentonite content on pellet compressive strength.

**Figure 12:**
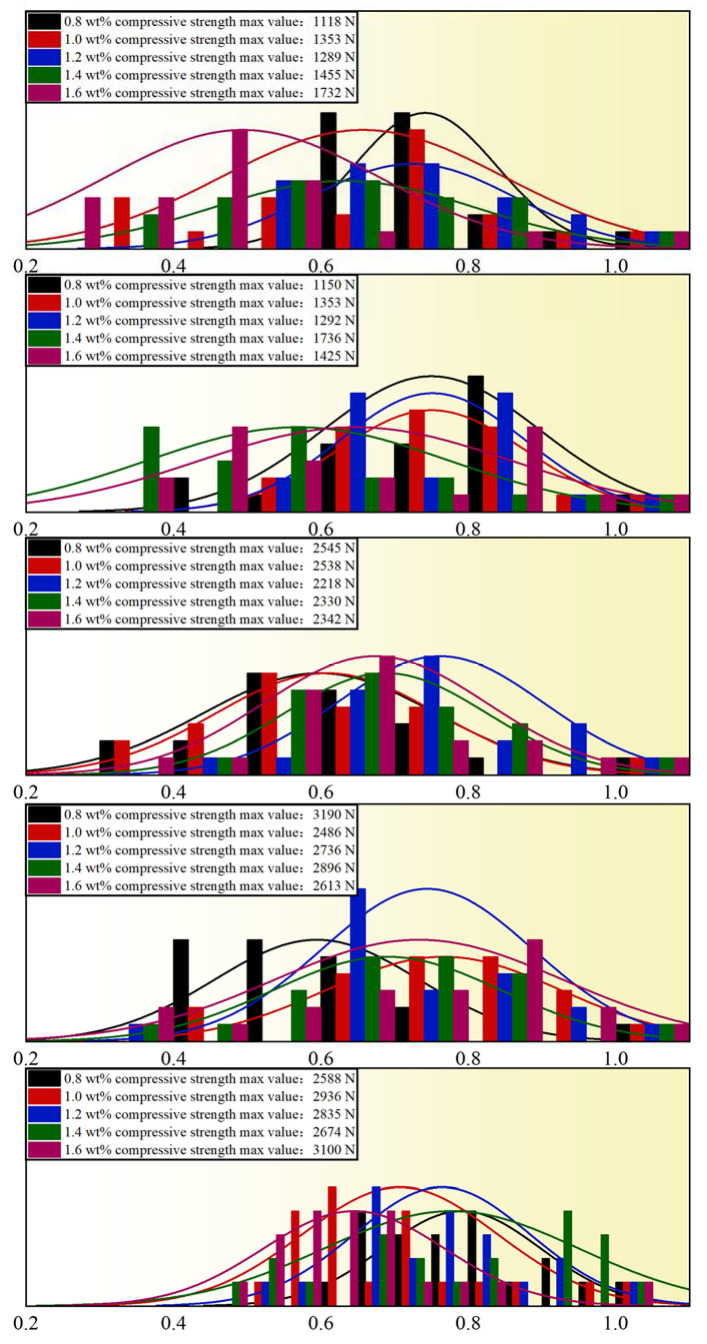
Normality test of pellet compressive strength under the influence of bentonite content.

The diameter of the pellets was controlled to be 9 mm, 10 mm, 11 mm, 12 mm, and 13 mm, and the calcination temperature was 1250 °C. The experiments were carried out in 25 groups to study the effect of the additional amount of bentonite on the compressive strength of the pellets, with 20 pellets for each group. The experimental results are shown in Figure 11. Twenty-five groups of experimental data were examined using the normality test and the linear analysis. Figure 12 displays the analysis findings. With the increased bentonite content, the pellet strength increases and decreases. When the bentonite content is below 1.2wt%, there is a positive correlation, and the effect is very significant; when the bentonite content is between 1.2wt% and 1.6wt%, the strength gradually decreases with the increase in bentonite content. It can be seen that the optimum addition amount of bentonite is about 1.2wt%.

**Figure 13:**
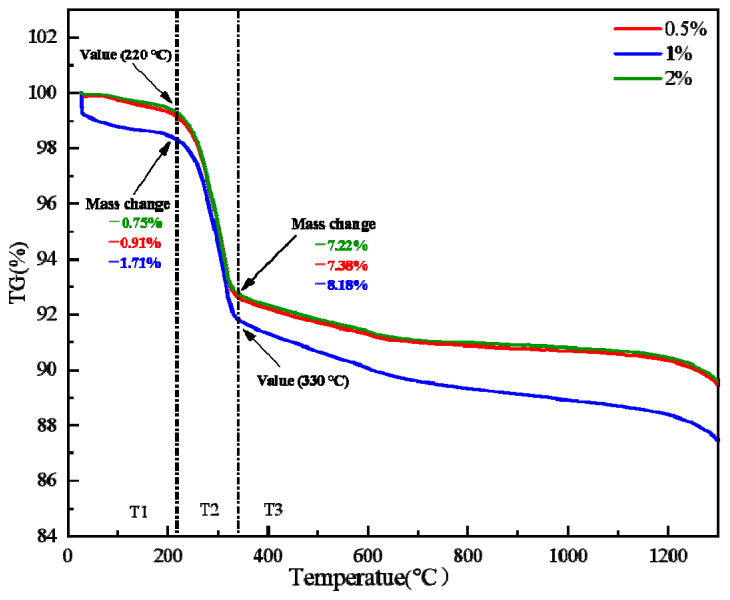
TG curve of limonite with different bentonite ratio.

**Figure 14:**
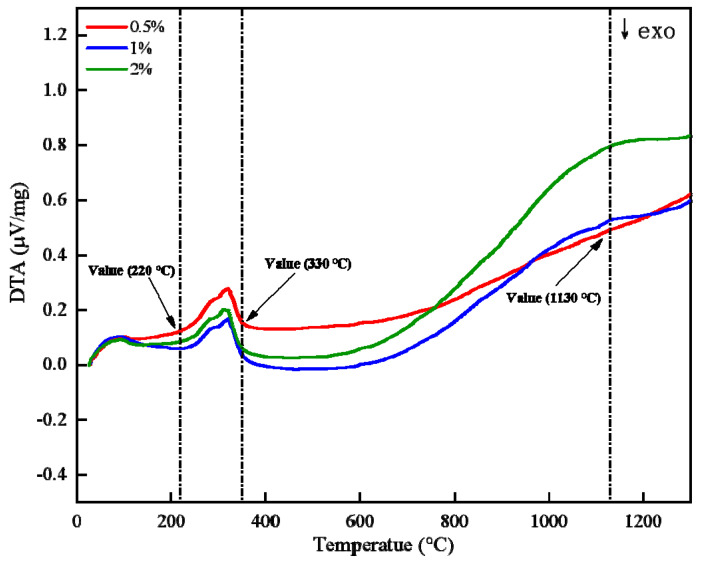
DTA curve of limonite with different bentonite ratio.

The desorption of crystal water from limonite is the main reason for the decrease in pellet strength [23,24,25]. To investigate the effect of bentonite on the desorption of crystal water, limonite powders with bentonite contents of 0.5wt%, 1wt%, and 2wt% were taken for differential thermal-thermogravimetric (TG-DTA) analysis. In an air atmosphere, the thermogravimetric (TG) and differential thermal (DTA) curves are shown in Figure 13 and Figure 14, respectively. Based on the thermogravimetric curve (Figure 13), it was discovered that the starting and ending temperatures of limonite crystal water desorption are basically the same when different contents of bentonite are added. It shows that the addition of bentonite has no significant effect on the desorption process of limonite crystal water. Analysis of the differential heat (DTA) curve (Figure 14) shows that the endothermic peak intensities of limonite at 200–400 °C are the same when the addition of bentonite is 0–2wt%. It shows that the addition of bentonite has no significant effect on the endothermic or exothermic effect of the desorption process of crystal water. Studies have demonstrated that the desorption performance of crystal water depends only on the degree of its binding to the ore [26]. The function of bentonite is mainly reflected in increasing the nucleation rate, green ball strength, and reducing the growth rate of green balls in ball making. It can be further inferred that the effect of bentonite on the compressive strength of pellets is mainly achieved by changing the pore structure.

### 3.3. Multifactorial Test

#### 3.3.1. Response Surface Design

The experiment was designed with three factors and three levels. The effects of three experimental parameters, including bentonite content, roasting temperature, and pellet diameter, on the compressive strength (Y) of pure limonite pellets were investigated. The strength of pure limonite was optimized to determine the best roasting process parameters using the response surface design by the design method of the statistical software. The factors and codes used in the experiment are shown in Table 4.

#### 3.3.2. Response Surface Method Design Results

The Box–Behnken experimental design was carried out using Statistical methods, and the experimental results are shown in Table 5. The quadratic model was used to perform regression fitting on the experimental results in Table 5. The mathematical prediction model of each influencing factor of the compressive strength of pure limonite pellets is established, as shown in Equation (3).

Y = −242092 + 386.2A − 140B − 11256C − 0.1610A^2^ − 56.6B^2^ − 415C^2^ + 1.490AB + 11.31AC − 176.7BC (3)

The statistical methods was used to perform variance analysis on the model, and the results are shown in Table 6. The F value in an ANOVA analysis is the ratio of the mean square between groups to the mean square within each group. The probability value under the corresponding F value is denoted by the *p* value. The presence of a *p* value less than 0.05 implies that the model item significantly impacts compressive strength. When the *p* value is less than 0.01, the factors substantially influence the compressive strength. It can be seen from Table 4 that the *p*-value of this model is less than 0.01, indicating that the selected experimental model fits well and has statistical significance. For the compressive strength of pure limonite pellets, The *p* value of A is greater than 0.05, indicating that A has no significant impact on compressive strength; the *p* value of B is less than 0.01, indicating that B has a very significant effect on compressive strength; and the *p* value of C is less than 0.05, indicating that C has a significant effect on compressive strength. Similarly, A^2^ has a tremendous impact on compressive strength in the square term, B^2^ has a significant impact on compressive strength, and C^2^ has no major effect on compressive strength. In the interaction term, the influence of AB and BC on compressive strength is negligible; however, the influence of AC on compressive strength is substantial. Among them, for the primary term of a single factor, the order of the influence of the three factors on compressive strength is: pellet diameter > bentonite ratio > calcination temperature.

**Figure 15:**
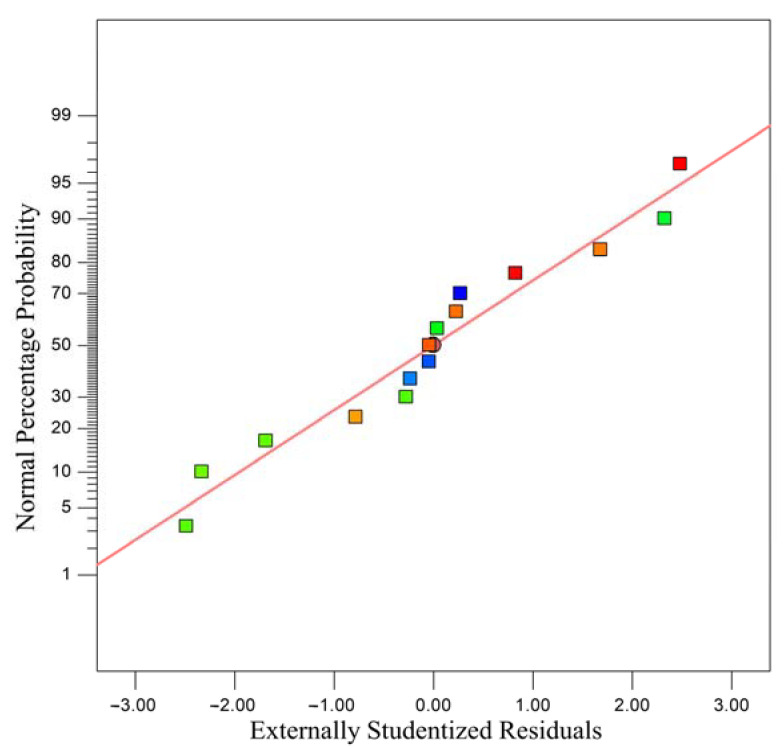
Residual normal probability map.

Figure 15 shows the normal distribution of residuals of the strength of pure limonite pellets. Random scattering patterns that do not follow a specific shape near a straight line indicate the high accuracy of regression modeling. The experimental points are approximately a straight line, indicating that the experimental selection model can be used to predict the experimental process within the normal range of the experimental residual distribution.

**Figure 16:**
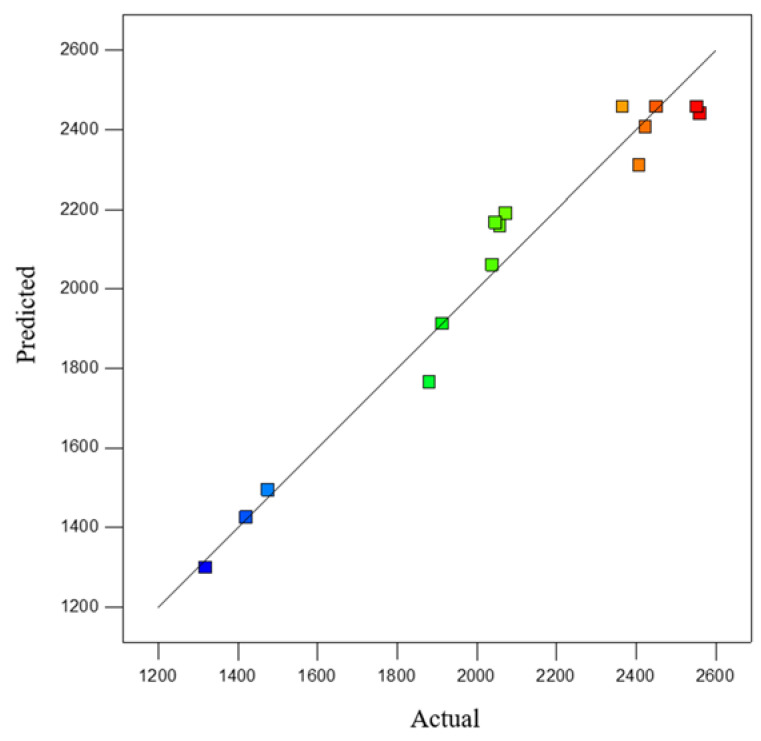
Relationship between experimental values of compressive strength of pellets and predicted values.

The comparison between the expected and experimental values of the compressive strength of pure limonite pellets is shown in Figure 16. The diagonal line in the figure means that the expected and experimental values are equal. The dots distributed around the slashes represent the predicted values relative to those experimentally obtained. It can be seen from Figure 16 that the experimental and predicted values are basically on both sides of this straight line, indicating that the experimental and predicted values have a high degree of fit.

#### 3.3.3. Response Surface Method Optimization

**Figure 17:**
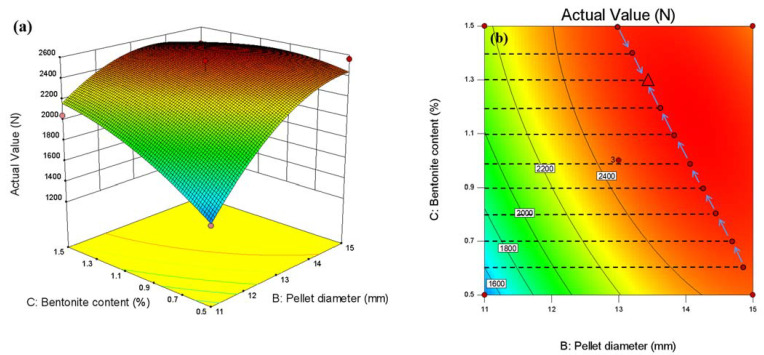
Response surface diagram between pellet diameter, bentonite content, and pellet compressive strength: (**a**) 3D response surface plot, (**b**) optimal parameter contour plot.

A 3D response surface plot shows the effect of model parameters on compressive strength. When the calcination temperature is 1300 °C, the response surface between the pellet diameter, the bentonite content, and the pellet compressive strength is shown in Figure 17a. It can be seen from the response surface in the figure that the experimental range is that the diameter of the pellet is 11–15 mm, and the ratio of bentonite is 0.5–1.5wt%. In the pellet diameter range of 11–13 mm, the compressive strength increases with the increase in pellet diameter; when the pellet diameter increases to 15 mm, the compressive strength decreases. In the bentonite ratio range of 0.5–1.3wt%, the compressive strength increases with the increase of the bentonite ratio, and the effect is significant on compressive strength. When the bentonite ratio is between 1.3wt% and 1.5wt%, the compressive strength declines as the bentonite ratio increases, essentially consistent with the results of the single-component experiment.

**Figure 18:**
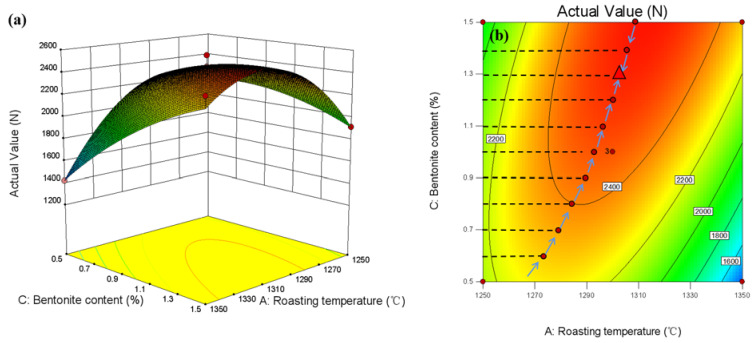
Response surface diagram between roasting temperature, bentonite content, and pellet compressive strength: (**a**) 3D response surface plot, (**b**) optimal parameter contour plot.

The response surface between the calcination temperature, the content of bentonite, and the compressive strength of the pellet when the diameter of the pellet is 13 mm is shown in Figure 18a. It can be seen from the curved surface in the figure that the experimental range is the calcination temperature of 1250 °C–1350 °C, and the ratio of bentonite is 0.5–1.5wt%. In the calcination temperature of 1250 °C–1300 °C, the compressive strength increases with the increase in calcination temperature. When the calcination temperature is between 1300 °C and 1350 °C, the compressive strength decreases with the increase in calcination temperature. In the bentonite ratio range of 0.5–1.3wt%, the compressive strength increases with the increase in bentonite ratio. In the bentonite ratio range of 1.3–1.5wt%, the compressive strength decreases with the rise in bentonite ratio, consistent with the univariate results.

**Figure 19:**
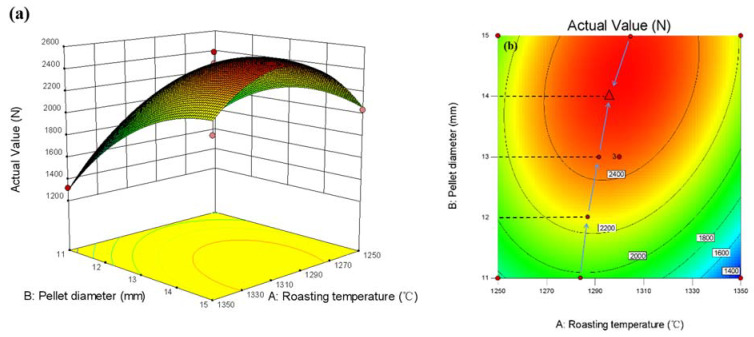
Response surface plot between pellet diameter, roasting temperature, and pellet compressive strength: (**a**) 3D response surface plot, (**b**) optimal parameter contour plot.

The response surface between pellet diameter, calcination temperature, and compressive strength of pellets when the bentonite content is 1wt% is shown in Figure 19a. It can be seen from the figure that the investigation conditions are 11–15 mm in diameter and 1250–1350 °C in calcination temperature. When the diameter of the pellet is 11–13 mm, with the increase in diameter, the compressive strength of the pellets is continuously improved. When the diameter reaches 15 mm, the compressive strength of the pellets shows a decreasing trend. When the investigated temperature is less than 1300 °C, the effect of temperature on activation energy is insignificant, and the impact on the improvement of compressive strength is weakened. When the temperature is more than 1300 °C, the effect of temperature on the compressive strength of the pellets is also insignificant, which is consistent with the single-factor experimental results.

Considering the interaction of three factors, the smallest ellipse is the highest point of the response surface in the two-dimensional contour plots. This oval-shaped 2D contour line indicates a significant interaction. Conversely, circular contour plots show weaker interactions. In addition, the higher the strength level of a contour line, the more interaction between the two factors. The contour lines of the optimum compressive strength process parameters are shown in Figure 17b, Figure 18b and Figure 19b. The interaction effect between the two independent variables can be easily understood from the figure. At the same time, the optimal level can be precisely located.

#### 3.3.4. Response Surface Method Optimization

The experimental parameters were investigated (calcination temperature 1250–1350 °C, pellet diameter 11–15 mm, bentonite content 0.5–1.5wt%), Comprehensive roasting temperature, roasting time, bentonite content, and other conditions are selected from the optimal experimental conditions optimized by the response surface software: The calcination temperature is 1310.61 °C, the pellet diameter is 13.9495 mm, and the bentonite content is 1.32828%, Its predicted compressive strength is 2570.3 N/piece. According to this optimal parameter condition, the pellet experiment is carried out again, and the experimental value of compressive strength is 2705 N/piece, its value is within the 95% confidence interval, and the relative error between the experimental value and the predicted value is only 5.2%, indicating that the model strength is relatively reliable.

## 4. Conclusions

1. The compressive strength of pure limonite pellets can reach 2700 N, which can be used in small blast furnaces and rotary hearth furnace processes.
2. Within the set factor range (calcination temperature 1250–1350 °C, bentonite content 0.5–1.5%, pellet diameter 11–15 mm), pellet diameter has the most significant influence on the compressive strength of pure limonite pellets, followed by the bentonite content, and the calcination temperature has no significant impact on the compressive strength of the pellets.
3. The roasting process of pure limonite pellets was optimized by statistical methods. Within the set factor range (calcination temperature 1250–1350 °C, bentonite content 0.5–1.5%, pellet diameter 11–15 mm), the optimized experimental conditions obtained are: bentonite content 1.32828%, calcination temperature 1310.61 °C, pellet diameter 13.9495 mm. The predicted value of pellet compressive strength is 2570.3 N/piece, and the experimentally verified value of pellet compressive strength is 2705 N/piece.
4. The content of bentonite does not affect the desorption process of limonite crystal water.

## Figures and Tables

**Table 1:** Experimental design.

Factor	Variable			Level		
		1	2	3	4	5
Roasting temperature/℃	A	1100	1150	1200	1250	1300
Pellet diameter/mm	B	9	10	11	12	13
Bentonite content/%	C	0.8	1.0	1.2	1.4	1.6

**Table 2:** Iron ore chemical composition.

w(TFe)	w(FeO)	w(SiO_2_)	w(S)	w(MnO)	w(TiO_2_)	w(Pb)	w(Zn)	w(K_2_O)	w(Na_2_O)	w(Cu)	w(V_2_O_5_)	Burning Loss
54.67	0.29	4.04	0.048	3.47	0.27	0.009	0.018	0.095	0.001	0.009	0.040	14.82

**Table 3:** The chemical composition of bentonite used in the experiment.

Moisture/%	Colloid Index/(%· (3g)^−1^)	Swelling Capacity/(mL·g^−1^)	Water Absorption/%	Methylene Blue Index/(g·(100g)^−1^)	Montmorillonite Mass Fraction/%
9.38	20.0	34.5	408	38.0	85.97

**Table 4:** Experimental factors and coding.

Factor	Variable	Level		
		−1	0	1
Roasting temperature/°C	A	1250	1300	1350
Pellet diameter/mm	B	11	13	15
Bentonite content/%	C	0.5	1.0	1.5

**Table 5:** Experimental results.

Run	Factor			Predicted Value/N	Actual Value/N
	Roasting Temperature/°C A	Pellet Diameter/mm B	Bentonite Content/% C		
1	−1	0	−1	2111.05	2059.00
2	0	1	−1	2392.50	2560.00
3	−1	−1	0	1717.20	1881.00
4	0	−1	−1	1444.30	1476.00
5	0	1	1	2359.00	2424.00
6	0	0	0	2408.50	2452.00
7	0	−1	1	2117.60	2047.00
8	0	0	0	2408.50	2366.00
9	1	−1	0	1247.20	1319.00
10	1	0	−1	1373.55	1421.00
11	−1	1	0	2014.00	2039.00
12	0	0	0	2408.50	2553.00
13	1	1	0	2140.00	2073.00
14	−1	0	1	1865.45	1915.00
15	1	0	1	2258.95	2408.00

**Table 6:** Variance analysis of the model.

Variable	Statistical Analysis				
	Sum of Squares	df	Mean Square	F-Value	*p*-Value
Model	2,251,335.15	9	250,148.35	13.26	0.0055
A	56,616.13	1	56,616.13	3.00	0.1438
B	703,891.12	1	703,891.12	37.31	0.0017
C	204,160.51	1	204,160.51	10.82	0.0217
AB	88,804.00	1	88,804.00	4.71	0.0822
AC	319,790.25	1	319,790.25	16.95	0.0092
BC	124,962.25	1	124,962.25	6.62	0.0498
A^2^	598,176.92	1	598,176.92	31.70	0.0024
B^2^	189,423.69	1	189,423.69	10.04	0.0249
C^2^	39,744.23	1	39,744.23	2.11	0.2064
Residual	94,341.25	5	18,868.25		
Lack of Fit	76,819.25	3	25,606.42	2.92	0.2652
Pure Error	17,522.00	2	8761.00		
Cor total	12,345,676.40	14			

## Data Availability

The data that support the findings of this study are available from the authors upon reasonable request.
